# A clinical score to predict recovery in end-stage kidney disease due to acute kidney injury

**DOI:** 10.1093/ckj/sfae085

**Published:** 2024-04-08

**Authors:** Silvi Shah, Jia H Ng, Anthony C Leonard, Kathleen Harrison, Karthikeyan Meganathan, Annette L Christianson, Charuhas V Thakar

**Affiliations:** Division of Nephrology and Hypertension, Department of Internal Medicine, University of Cincinnati, Cincinnati, OH, USA; Division of Kidney Diseases and Hypertension, Department of Medicine, Donald and Barbara Zucker School of Medicine at Hofstra/Northwell, Great Neck, NY, USA; Department of Environmental Health, University of Cincinnati, Cincinnati, OH, USA; Division of Nephrology and Hypertension, Department of Internal Medicine, University of Cincinnati, Cincinnati, OH, USA; Department of Environmental Health, University of Cincinnati, Cincinnati, OH, USA; Department of Environmental Health, University of Cincinnati, Cincinnati, OH, USA; Division of Nephrology and Hypertension, Department of Internal Medicine, University of Cincinnati, Cincinnati, OH, USA; Wellcome-Wolfson Institute of Experimental Medicine, School of Medicine, Dentistry and Biomedical Sciences, Queen's University Belfast, Belfast, Northern Ireland

**Keywords:** AKI, dialysis, end-stage kidney disease, kidney failure, recovery

## Abstract

**Background:**

Acute kidney injury (AKI) is a major contributor to end-stage kidney disease (ESKD). About one-third of patients with ESKD due to AKI recover kidney function. However, the inability to accurately predict recovery leads to improper triage of clinical monitoring and impacts the quality of care in ESKD.

**Methods:**

Using data from the United States Renal Data System from 2005 to 2014 (*n* = 22 922), we developed a clinical score to predict kidney recovery within 90 days and within 12 months after dialysis initiation in patients with ESKD due to AKI. Multivariable logistic regressions were used to examine the effect of various covariates on the primary outcome of kidney recovery to develop the scoring system. The resulting logistic parameter estimates were transformed into integer point totals by doubling and rounding the estimates. Internal validation was performed.

**Results:**

Twenty-four percent and 34% of patients with ESKD due to AKI recovered kidney function within 90 days and 12 months, respectively. Factors contributing to points in the two scoring systems were similar but not identical, and included age, race/ethnicity, body mass index, congestive heart failure, cancer, amputation, functional status, hemoglobin and prior nephrology care. Three score categories of increasing recovery were formed: low score (0–6), medium score (7–9) and high score (10–12), which exhibited 90-day recovery rates of 12%, 26% and 57%. For the 12-month scores, the low, medium and high groups consisted of scores 0–5, 6–8 and 9–11, with 12-month recovery rates of 16%, 33% and 62%, respectively. The internal validation assessment showed no overfitting of the models.

**Conclusion:**

A clinical score derived from information available at incident dialysis predicts renal recovery at 90 days and 12 months in patients with presumed ESKD due to AKI. The score can help triage appropriate monitoring to facilitate recovery and begin planning long-term dialysis care for others.

## INTRODUCTION

Acute kidney injury (AKI), predominantly caused by acute tubular necrosis (ATN), is frequent during acute hospitalizations and is associated with an increased risk of chronic kidney disease (CKD) and end-stage kidney disease (ESKD), and a higher risk of cardiovascular events and mortality [1–3]. Kidney failure caused by AKI has a 28% greater mortality risk than other causes of kidney failure [4]. Morbidity, mortality and costs of care are among the highest during the transition to kidney failure, particularly in the first year of dialysis care [5]. Therefore, maximizing kidney recovery in patients with ESKD due to AKI is critical.

Currently, the approach to outpatient dialysis care for patients with ESKD due to AKI is widely variable [6]. This is due to the difficulty in identifying those who will recover and the lack of evidence on the optimal management for this population. Among patients with ESKD due to AKI, 35% experience kidney recovery. Within this subset of those who had kidney recovery, up to 95% recovered within 12 months [4]. Thus, the AKI group needs a different outpatient dialysis treatment plan that promotes recovery, reduces kidney re-injury and monitors kidney recovery. These practices are resource intensive, and require proper identification and risk stratification to predict who will most likely recover to optimize good kidney outcomes.

Several studies have identified independent predictors of kidney recovery in patients with ESKD due to AKI [4, 7–10]. However, the cumulative effect of several predictors on kidney recovery is unknown, limiting their utility in clinical practice. A recent study showed that, among patients with kidney failure due to AKI, sex, race/ethnicity and vascular access were important factors associated with kidney recovery [4]. To perform an optimal intervention, we need to risk-stratify those with AKI and the simultaneous effect of multiple risk factors on kidney recovery [11, 12]. This study aimed to develop and validate a clinical score that predicts kidney recovery in patients with ESKD due to AKI after accounting for the effect of various risk factors associated with recovery, using the largest national administrative dataset in the USA.

## MATERIALS AND METHODS

### Study population

**Figure 1:**
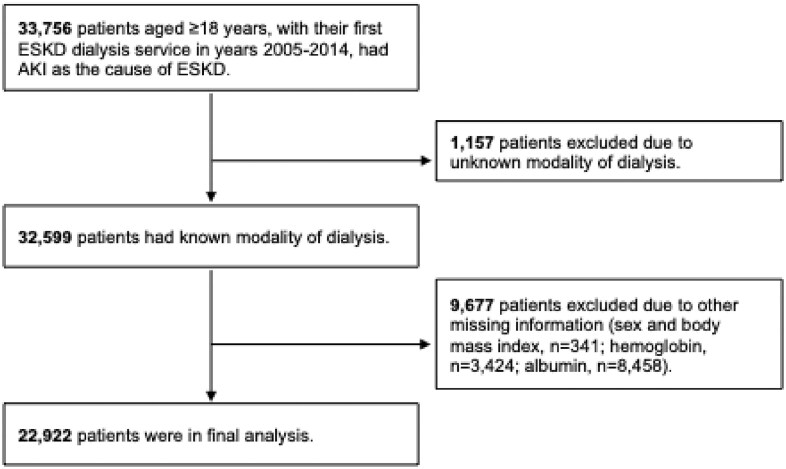
Study cohort derivation.

The University of Cincinnati Institutional Review Board committee deemed the study exempted due to the use of de-identified data. We studied 22 922 adults listed in the United States Renal Data System (USRDS), who initiated dialysis between 1 January 2005 and 31 December 2014, with AKI as the presumed cause of ESKD and who were at least 18 years old at dialysis initiation. AKI was defined as “tubular necrosis” (code 5836 or 5836A) as annotated by the provider on the CMS-form 2728. Patients with missing information on sex, dialysis modality, dialysis access, body mass index, albumin or hemoglobin were excluded. Figure 1 illustrates the study cohort derivation.

The USRDS patients file was used to obtain information on the date of ESKD incidence (identified by date of first ESKD service), cause of ESKD, age, race/ethnicity, sex, date of death, first kidney transplant date and the initial dialysis modality. The Centers for Medicare and Medicaid Services (CMS) form-2728 data in the USRDS Medevid file was used to obtain information on body mass index, comorbidities, pre-dialysis nephrology care, history of nursing home, laboratory data and functional status. Kidney recovery was determined from the USRDS Rxhist file [13].

### Outcomes and covariates

The primary outcome was kidney recovery in patients with kidney failure due to AKI at 90 days and 12 months after dialysis initiation. Patients who died or had a kidney transplant during the 90-day or 12-month period before recovery were deemed not recovered for that period. The following covariates were included to develop a 90-day and 12-month recovery score: the age at dialysis initiation, race/ethnicity, sex, body mass index, comorbidities, poor functional status, history of nursing home residence, laboratory data (serum hemoglobin and serum albumin within 45 days prior to most recent ESKD episode) and pre-dialysis nephrology care. The following comorbidities were included: diabetes mellitus, congestive heart failure, hypertension, cancer, amputation, cerebrovascular accident/transient ischemic attack, chronic obstructive pulmonary disease, atherosclerotic heart disease or other cardiac disease and peripheral vascular disease. Poor functional status was defined by any of the three comorbid conditions as specified in form CMS-2728: inability to ambulate, inability to transfer or need for assistance with daily activities [14]. All covariates were classified as categorical variables (Table 1). We chose the above covariates based on prior literature studying predictors of kidney recovery [4, 7–10, 12, 15].

**Table 1:** Bivariate comparisons of patient characteristics by recovered vs not recovered kidney function at 90 days and at 12 months, for predictors used in multivariable models, and for mortality.

		90 day recovery		12 month recovery	
Characteristics	All, *n* = 22 922	Yes, *n* = 5524 (24.1%)	No, *n* = 17 398 (75.9%)	Yes, *n* = 7686 (33.5%)	No, *n* = 15 236 (66.5%)
Demographics					
Age, years^a^	66.3 (14.3)	60.9 (15.4)	68.0 (13.6)	61.9 (15.0)	68.5 (13.5)
18–29	1.5	3.3	1.0	2.6	1.0
30–39	3.6	7.0	2.5	6.0	2.3
40–49	7.6	12.2	6.2	11.5	5.7
50–59	16.8	21.1	15.4	21.0	14.7
60–69	24.6	24.0	24.8	24.9	24.5
70–79	26.9	21.8	28.5	22.5	29.1
≥80	19.0	10.6	21.7	11.5	22.8
Sex					
Women	58.4	63.0	56.9	62.8	56.1
Men					
Race/ethnicity					
White	75.5	78.9	74.4	77.9	74.2
Asian	1.8	1.6	1.9	1.6	1.9
Black	14.5	12.0	15.3	12.4	15.6
Hispanic	7.7	7.1	7.9	7.6	7.7
Native American	0.5	0.5	0.5	0.6	0.5
Body mass index, kg/m^2a^	29.1 (8.4)	29.9 (8.2)	28.9 (8.5)	29.7 (8.2)	28.8 (8.5)
<18.5	4.4	2.9	4.8	3.2	4.9
18.5–<25	31.6	27.1	33.0	28.0	33.4
≥25–30	27.4	29.3	26.7	28.7	26.7
>30	36.7	40.7	35.5	40.1	35.1
Comorbidities					
Congestive heart failure	35.5	23.0	39.4	24.5	41.0
Atherosclerotic heart disease + others	43.5	34.5	46.4	36.4	47.1
Hypertension	72.9	66.5	75.0	68.1	75.3
Diabetes mellitus	39.7	33.9	41.6	35.4	41.9
Cancer	14.1	11.8	14.8	11.8	15.3
Amputation	2.9	1.9	3.3	2.3	3.2
Peripheral vascular disease	15.0	11.1	16.2	12.1	16.4
Cerebrovascular accident/transient ischemic attack	10.0	7.1	10.9	7.7	11.2
Chronic obstructive pulmonary disease	14.9	11.5	16.0	11.9	16.4
Poor functional status	26.1	16.2	29.3	18.4	30.0
Laboratory values					
Albumin, g/dL^a^					
<3.5	2.8 (0.8)	2.8 (0.8)	2.8 (0.8)	2.8 (0.8)	2.8 (0.8)
<3.5	81.4	82.4	81.0	82.3	80.9
≥3.5	18.6	17.5	19.0	17.8	19.1
Hemoglobin, g/dL^a^	10.0 (1.8)	10.4 (2.1)	9.9 (1.7)	10.3 (2.0)	9.8 (1.7)
<11	76.3	68.6	78.7	70.6	79.1
11–12	13.1	14.6	12.6	14.2	12.5
>12	10.7	16.8	8.7	15.2	8.3
Care					
History of nursing home	16.7	10.9	18.6	12.0	19.1
Prior nephrology care					
None	62.1	75.2	57.9	73.8	56.1
≤12 months	18.5	11.3	20.8	11.9	21.9
>12 months	7.6	3.3	8.9	3.7	9.5
Unknown	11.9	10.3	12.4	10.5	12.5

Significance tests were Chi-squares for categorical variables and Wilcoxon tests for continuous variables. All comparisons of recovered vs not (either 90-day or 12-month) have *P*-value <.001 except for albumin split at 3.5: *P*-value = .02 for 90-day recovery and *P*-value = .01 for 12-month recovery.

Reported in %. ^a^Mean ± standard deviation

### Statistical analysis

Summary statistics on all measures are reported as mean (standard deviation) or *N* (%) for the overall sample and relevant subgroups such as recovered and non-recovered patients. The scoring systems predicting 90-day and 12-month recovery were developed independently using methods described in Sullivan *et al*. [16]. For each scoring system, a multivariable, non-parsimonious logistic model predicted recovery by the end of the time period, with patients dying or receiving a kidney transplant during the time period, before recovering, deemed not recovered. Each predictive factor in the model had its reference level set at the level with the highest recovery rate in the simple bivariate analyses. The logistic parameter estimates from the model were then multiplied by 2 and rounded to the nearest integer. Because the reference level of each predictive factor was the level most associated with recovery, estimates for the other levels of the factor were always negative. The negative integer scores were then adjusted by adding to each an integer large enough to make the most negative score zero, thus resulting in scores that were always positive. The score of zero was the level of a factor associated with the lowest rate of recovery. Each patient's total recovery score was then the sum of the points obtained from the 18 predictive factors.

To evaluate the loss of predictive information when going from a full logistic regression using the original predictors to the single recovery score generated for each patient, we compared the area under the receiver operating characteristic curves (AUROC), also known as the c-statistic, from the 18 factors predicting kidney recovery to the one recovery score predicting recovery. The loss was calculated by subtracting 0.5 from each c-statistic (because a value of 0.5 indicates no predictive power), following which the % reduction was calculated [17].

Because we anticipated logistic parameter estimates that would be close to the boundaries at which they would or would not result in a point in the scoring system (an estimate of −0.24 leads to no points for that factor, whereas −0.26 leads to a point), we conducted two different internal validation processes to evaluate the optimism (overfitting) in the power of the scoring systems to predict kidney recovery [18]. In the first method, one patient at a time was removed from the sample and the scoring system was re-generated using all the remaining patients (jackknifing), and that scoring system was then used to arrive at a score for the removed patient. This was repeated for all 22 922 patients, so each had a cross-validation score from a system developed without that patient. Those scores were used to predict recovery in the same fashion as the original scores, and the difference in the two resulting c-statistics was optimism due to overfitting. In the second method, we generated 500 bootstrap samples of size 22 922 and, using each developed the scoring system using the original methods. Each bootstrap-based system was then applied to the bootstrap sample from which it was derived and to the original sample, logistic regressions predicting recovery from the recovery scores were estimated, and the regression-based c-statistics were obtained from each bootstrap sample and each of the 500 systems applied to the original sample. The average difference between c-statistics from the bootstrap sample and that obtained from the original sample was the estimate of optimism due to overfitting.

Finally, we collapsed both scoring system scales into three categories of low, medium and high risk, using the proportions of patients in each category and the recovery rates to form risk groups deemed useful to the healthcare providers making treatment decisions. All analyses were conducted using SAS version 9.4 (SAS Institute, Cary, NC, USA).

## RESULTS

### Baseline characteristics of the study cohort

The study cohort consisted of 22 922 patients with ESKD due to AKI (Fig. 1). Patients’ mean age ± standard deviation was 66 ± 14 years, 58% were men and 76% were of white race/ethnicity. The mean body mass index was 29 ± 8 kg/m^2^. Kidney recovery within 90 days occurred in 24% of patients, and recovery within 12 months occurred in 34% of patients. Fifteen percent of patients died within 90 days of starting dialysis, and 35% died within 12 months. Univariate factors associated with kidney recovery within 90 days and within 12 months are shown in Table 1. Factors most strongly associated with recovery were largely the same for both recovery periods (90 days and 12 months). All variables, including demographics, comorbid conditions, laboratory values and prior nephrology care, were significantly associated with kidney recovery (either 90-day or 12-month, *P*-value <.001; except for albumin split at 3.5: *P*-value = .02 for 90-day recovery and *P*-value = .01 for 12-month recovery).

Table 2 shows the coefficients from the multivariable logistic regressions predicting both 90-day and 12-month recovery and the scoring system points derived from those coefficients. The 90-day recovery scoring system included points for a younger age, higher body mass index, white or Asian race, absence of the comorbidities of congestive heart failure, amputation, cancer or poor functional status, hemoglobin >12 g/dL, and unknown or no prior nephrology care. The factors receiving points for the 12-month kidney recovery score were similar to the 90-day factors except for the addition of Hispanic ethnicity, the exclusion of amputation and a different weighting for some age groups.

**Table 2:** Logistic regressions predicting recovery of kidney function within 90 days and within 12 months, with resulting points for recovery scores.

	90-day recovery			12-month recovery		
Characteristics	Parameter estimate	Estimates ×2, rounded	Final, positive recovery points	Parameter estimate	Estimates ×2, rounded	Final, positive recovery points
Demographics						
Age, years						
18–29 (reference)	0	0	3	0	0	3
30–39	−0.2322	0	3	−0.0676	0	3
40–49	−0.4754	−1	2	−0.2111	0	3
50–59	−0.7469	−1	2	−0.4566	−1	2
60–69	−0.9699	−2	1	−0.6791	−1	2
70–79	−1.1483	−2	1	−0.8977	−2	1
≥80	−1.5458	−3	0	−1.2815	−3	0
Sex						
Male (reference)	0	0	0	0	0	0
Female	−0.1797	0	0	−0.2208	0	0
Race/ethnicity						
White (reference)	0	0	1	0	0	1
Asian	−0.2424	0	1	−0.2419	0	1
Black	−0.4915	−1	0	−0.4676	−1	0
Hispanic	−0.3126	−1	0	−0.2027	0	1
Native American	−0.5059	−1	0	−0.3372	−1	0
Body mass index, kg/m^2^						
<18.5	−0.7228	−1	0	−0.6132	−1	0
18.5–<25	−0.3384	−1	0	−0.3068	−1	0
≥25–30	−0.0564	0	1	−0.0657	0	1
>30 (reference)	0	0	1	0	0	1
Comorbidities						
Congestive heart failure						
No (reference)	0	0	1	0	0	1
Yes	−0.4776	−1	0	−0.5072	−1	0
Atherosclerotic heart disease + others						
No (reference)	0	0	0	0	0	0
Yes	−0.0556			−0.0288		
Hypertension						
No (reference)	0	0	0	0	0	0
Yes	−0.1167	0	0	−0.0856	0	0
Diabetes mellitus						
No (reference)	0	0	0	0	0	0
Yes	−0.1431	0	0	−0.1253	0	0
Cancer						
No (reference)	0	0	1	0	0	1
Yes	−0.2535	−1	0	−0.3121	−1	0
Amputation						
No (reference)	0	0	1	0	0	0
Yes	−0.3208	−1	0	−0.2061	0	0
Peripheral vascular disease						
No (reference)	0	0	0	0	0	0
Yes	−0.0253	0	0	0.019	0	0
Cerebrovascular accident/transient ischemic attack						
No (reference)	0	0	0	0	0	0
Yes	−0.125	0	0	−0.1114	0	0
Chronic obstructive pulmonary disease						
No (reference)	0	0	0	0	0	0
Yes	−0.0958	0	0	−0.1212	0	0
Poor functional status						
No (reference)	0	0	1	0	0	1
Yes	−0.5436	−1	0	−0.4406	−1	0
Laboratory values						
Albumin, g/dL						
<3.5 (reference)	0	0	0	0	0	0
≥3.5	−0.1811	0	0	−0.1469	0	0
Hemoglobin, g/dL						
<11	−0.5848	−1	0	−0.515	−1	0
11–12	−0.3025	−1	0	−0.2777	−1	0
>12 (reference)	0	0	1	0	0	1
Care						
History of nursing home						
No (reference)	0	0	0	0	0	0
Yes	−0.0862	0	0	−0.103	0	0
Prior nephrology care						
None (reference)	0	0	2	0	0	2
0–12 months	−0.8025	−2	0	−0.8303	−2	0
>12 months	−1.0695	−2	0	−1.0653	−2	0
Unknown	−0.383	−1	1	−0.4018	−1	1

Parameter estimates are from multivariable logistic regressions with the category within each factor most likely to recover as the reference group. Estimates ×2 rounded are double the estimates rounded to the nearest integer. Final positive points are the estimates ×2 rounded, with points added within each factor to make all points zero or positive.

**Figure 2:**
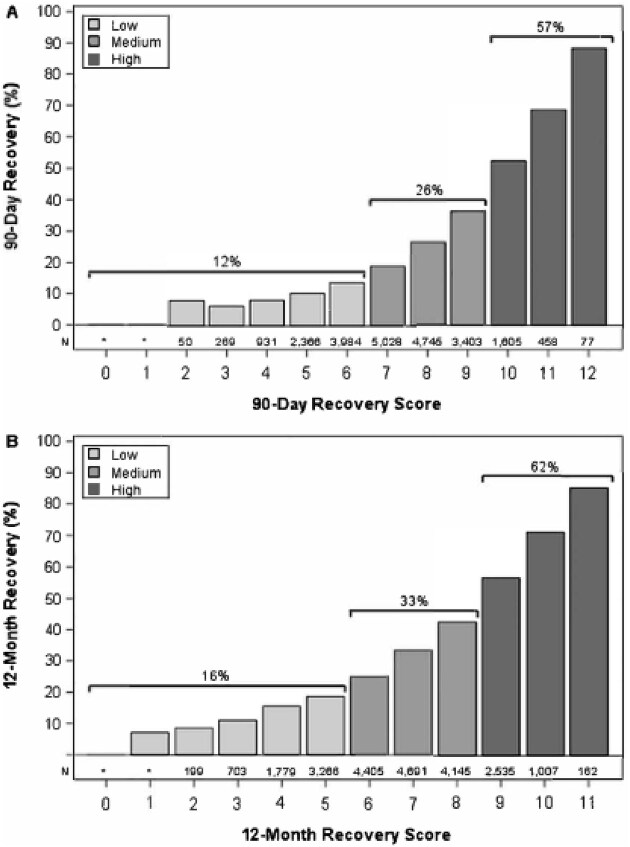
Recovery rates of patients at different recovery score levels, with total number of patients at each level, and with collapses into low-, medium- and high-risk groups, for (**A**) recovery within 90 days post-dialysis initiation, and (**B**) recovery within 12 months post-dialysis initiation.

Total recovery scores possible for 90-day recovery ranged from 0 to 12, and for 12-month recovery, from 0 to 11 (Table 3A). For 90-day recovery, patients that eventually recovered had mean recovery scores of 8.2 ± 1.7, while the non-recovered patients had mean recovery scores of 6.9 ± 1.6. As shown in Fig. 2, observed recovery rates increased steadily with increases in the recovery scores, with 88% recovered among patients with a maximum recovery score of 12 and <10% at any score level of 4 or less. The consistent alignment of scores and recovery rates was broken only between score levels of 2 and 3, levels at which the numbers of patients were small. For 12-month recovery, the mean scores of recovered and non-recovered patients were 7.5 ± 1.7 and 6.3 ± 1.7, respectively; 85% of patients with a maximum score of 11 recovered, and the recovery rate declined steadily to under 10% at score levels 2 and below (Table 3B).

**Table 3A:** Factors predicting recovery within 90 days of dialysis initiation, and within 12 months of dialysis initiation.

	Within 90 days of dialysis initiation	Within 12 months of dialysis initiation
(i) Age (years)		
18–29	3	3
30–39	3	3
40–49	2	3
50–59	2	2
60–69	1	2
70–79	1	1
(ii) Race/ethnicity		
Asian	1	1
Hispanic	No score	1
White	1	1
(iii) Body mass index ≥25 kg/m^2^	1	1
(iv) No history of congestive heart failure	1	1
(v) No history of cancer	1	1
(vi) No history of amputation	1	No score
(vii) No poor functional status	1	1
(viii) Hemoglobin >12 g/dL	1	1
(ix) No prior nephology care	2	2
Unknown prior nephology care	1	1
Maximum possible points	12	11

**Table 3B:** Proportions of patients recovered from AKI within 90 days and within 12 months at various recovery score levels.

	Within 90 days of dialysis initiation	Within 12 months of dialysis initiation
Recovery score	% Recovered	% Recovered
0	n/a^a^	0.0
1	0.0	7.1
2	8.0	8.5
3	6.0	11.0
4	8.1	15.5
5	10.1	18.6
6	13.5	25.0
7	18.7	33.3
8	26.4	42.4
9	36.4	56.5
10	52.3	71.1
11	68.6	85.2
12	88.3	

a There were no patients with a score of 0.

Minimum score, 0; maximum score, 12 for 90-day and maximum score, 11 for 12-month. For the 90-day scores; the low-, medium- and high-risk groups consisted of scores 0–6, 7–9 and 10–12, and recovery rates of 12%, 26% and 57%. For the 12-month scores; the low-, medium- and high-risk groups consisted of scores 0–5, 6–8 and 9–11, and recovery rates of 16%, 33% and 62%.

All the information is available from form-2728. Poor functional status includes any of the following: inability to ambulate, inability to transfer or needing assistance with activities of daily living.

Three collapsed score categories for 90-day recovery were also formed: low score (0–6), medium score (7–9) and high score (10–12). Those groups contained 33%, 58% and 9% of patients, respectively, and patients within them had 90-day recovery rates of 12%, 26% and 57%, respectively. Similarly, for the 12-month recovery scores, groupings of low score (0–5), medium score (6–8) and high score (9–11) contained 26%, 58% and 16% of the patients, respectively, and had 12-month recovery rates of 16%, 33% and 62%. (Fig. 2).

### Predictive powers of the scoring systems

**Figure 3:**
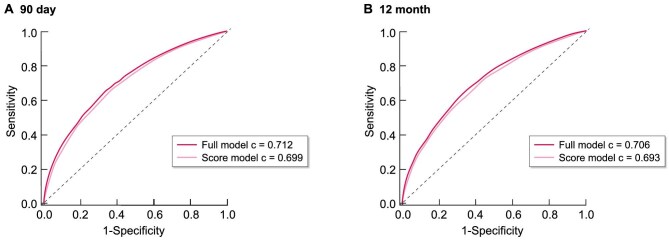
ROC curves from the four logistic models predicting renal recovery in patients with kidney failure due to AKI, at (**A**) 90 days post-dialysis initiation, and (**B**) 12 months post-dialysis initiation. The graphs show that by going from the original logistic regression predicting recovery to the scoring system scores, very little predictive power is lost. *The “full models” use the full set of covariates predicting recovery, while the resulting “score models” use the resulting scoring system recovery scores to predict the same. The c-statistic is the area under each ROC curve.

To assess the loss of predictive power as measured by changes in the area under the ROC curves when going from the original logistic regressions to the derived scores, we analyzed and compared ROC curves from both the original logistic regression models and the derived score models. For the prediction of 90-day kidney recovery, moving from the full logistic model to the score model led to a shrinkage in the c-statistic (area under the ROC curve) from 0.712 to 0.699, or 6.3% of the value above 0.5 (an area under the ROC curve of 0.5 indicates no prediction). For the prediction of 12-month kidney recovery, the shrinkage was from 0.706 to 0.693, or 6.4% of the value above 0.5, showing that the derived score model performed similarly to the original logistical regression model. Figure 3 shows the ROC curves.

### Internal validation of scoring system (bootstrap and jackknife methods)

For estimates of optimism in the c-statistic due to overfitting, the 90-day recovery score for the jackknife method was 0.00234, or 1.2% of prediction above c = 0.5. For the bootstrap estimate, it was 0.0021, or 1.1% of effective predictive power. For the 12-month score and the jackknife method, the optimism was 0.001615, or 0.8% of prediction above c = 0.5. For the bootstrap method of estimating the optimism, it was 0.0018, or 0.9%; Pearson correlations between the original patient scores and the scores derived by the jackknife cross-validation method was r = 0.99941 for the 90-day recovery scores and r = 0.99953 for the 12-month recovery scores. These internal validation results indicate that any overfitting in the modeling processes was very small and minimally impacted recovery scores.

## DISCUSSION

In one of the largest studies derived from a national cohort, we report the development of clinical risk score derived from information readily available at incident dialysis to predict kidney recovery at 90 days and 12 months in patients with ESKD due to AKI. To achieve adequate power and generalizability, the present study analyzed a large cohort of patients well represented by differences in sex and race and includes all patients with ESKD due to AKI with varying degrees of risk, thus making it more clinically applicable. Our data indicate that the proposed clinical score can be used in predicting kidney recovery in ESKD due to AKI.

In this study, we propose two scoring systems to predict AKI recovery at 90 days and 12 months, and we showed that increasing score numbers confer a higher chance of kidney recovery. The 90-day AKI recovery scores variables include age, race/ethnicity, body mass index, history of congestive heart failure, history of cancer, history of amputation, poor functional status, hemoglobin level and prior nephrology care. The 12-month AKI recovery score has the same variables as the 90-day recovery score, apart from the “amputation” variable. The scoring system's ability to identify patients with low, medium and high changes in kidney recovery may aid with patient stratification, particularly in informing monitoring, treatment plans and follow-up decisions. Additionally, these variables can be ascertained easily through patient charts and history-taking, increasing the clinical utility of the scoring system.

Prior literature has shown that demographic variables such as age and race are associated with kidney recovery [4, 8, 12, 19–25]. In our study, older age was associated with a lower chance of kidney recovery, potentially due to physiologic changes with aging kidneys and age-related cardiovascular diseases such as hypertension and microvascular disease [21, 26–29]. We also found that compared with white patients, all other racial categories were less likely to experience recovery. Similarly, Foley *et al*. showed 31% and 21% lower likelihood of kidney recovery of the Black population vs white population, and Hispanic ethnicity vs non-Hispanic ethnicity, respectively [25]. These disparities may stem from differences in AKI management, such as late initiation of dialysis, which may reflect reduced access to care [19–23]. Including the age, race and ethnicity variable in the AKI recovery scores will provide better prognostication of an individual's chance of kidney recovery and, in turn, provide better education for self-management.

Our study emphasized the impact of comorbid conditions on kidney recovery, with conditions such as lower body mass index, heart failure, cancer, amputation, poor functional status and low hemoglobin levels contributing to lower kidney recovery rates. Similar to what was shown in our study, the presence of heart failure reduces the chance of kidney recovery after AKI [8, 11, 30]. Patients with congestive heart failure are more likely to have poor cardiac output or systematic venous congestion, which reduces renal perfusion and thereby decreases the chance of renal recovery [31, 32]. The presence of cancer and low hemoglobin could reflect the severity of illness or treatment surrounding the AKI event [8, 15, 33, 34]. Poor functional status and amputation could reflect the patient's baseline physical reserve or a surrogate marker of illness severity surrounding the AKI event [35, 36]. Additionally, patients with baseline poor functional status and frailty were found to be more likely to develop AKI that were higher in severity, potentially leading to a lower rate of recovery [35, 36].

Not receiving prior nephrology care and unknown prior nephrology care were independent predictors of kidney recovery. Previous studies have shown that early nephrology referral confers better kidney management and delay in the progression of kidney failure [37]. However, since our study population is among those who developed kidney failure from AKI, it is likely that many patients did not require nephrology care prior to the AKI episode. Given that the USRDS data begins with incident dialysis information, we could not ascertain the level of CKD prior to patients experiencing AKI. To that effect, the variable defining pre-ESKD nephrology care may be considered as a surrogate for receiving care for CKD before incident ESKD. This may explain our observation that pre-ESKD nephrology care was associated with a lower likelihood of kidney recovery. Regarding “unknown prior nephrology care,” the variable likely contains a mix of people with and without nephrology care, thus putting the group at the intermediate probability of recovery.

We have shown that simple scoring systems using integer weights based on multivariable logistic regressions can predict recovery from AKI at both 90 days and 12 months. The loss of information from the original regressions to the scoring systems is minimal, as is any overfitting in the model estimation and scoring system construction processes. Despite the use of administrative data to predict kidney recovery in our study, the performance of the models was fair, with AUC within 0.70. As a comparison, other studies using biomarkers to predict kidney recovery had AUC in the range of 0.66–0.79 [38–40], whereas studies using granular data from the electronic health record had AUC ranging between 0.73 and 0.83 [41, 42].

The proposed AKI recovery scores may enable the identification of people with low, medium and high chances of recovery, both at the 90-day and 12-month mark. This is important because the mortality for patients with dialysis-dependent AKI is the highest during the first year of dialysis, and those who get off dialysis have better clinical and patient-centered outcomes [4]. Dialysis unit treatments are currently protocolized for patients with ESKD, not specifically for patients with AKI. The ability to stratify patients with a higher chance of kidney recovery may allow clinicians to decide which patient would require frequent monitoring for kidney recovery and for whom to implement dialysis treatments that are catered towards promoting kidney recovery and avoiding re-injury (lower ultrafiltration rates, avoidance of intradialytic hypotension).

The scoring system developed in this study focuses on predicting kidney recovery in patients with ATN, and its application to other causes of AKI may be limited. Other etiologies of AKI, such as acute interstitial nephritis and glomerulonephritis, would have distinct pathophysiological processes, risk factors and recovery, which our scoring system will not fully capture. While this scoring system is a useful tool for ATN, the most common cause of AKI, its application should be tailored considering the underlying cause of AKI. Any use beyond ATN warrants cautious interpretation.

Our analysis has limitations inherent to using administrative registries, such as a lack of ability to demonstrate exact causality and possible misclassification due to physician bias in determining ESKD due to AKI from the CMS-form 2728. The study chose the designation of ESKD due to “tubular necrosis,” because it is the most common cause of AKI, and contributor to presumed ESKD. We acknowledge that there may be other causes of clinically apparent AKI (for example, interstitial or glomerular disorders), but including them would have added more heterogeneity in terms of natural history and treatment. This should be noted while planning clinical monitoring for recovery. This form is designed to capture data for ESKD; thus, it also lacks pre-AKI data. We acknowledge that the study period overlaps with the time when dialysis-dependent AKI became an allowable condition to be dialyzed in chronic dialysis units, and thus our recovery rates may be reflective of that subgroup as well. However, a national sample derived from a validated USRDS database allows us to study patterns and predictors of recovery in a robust fashion. The USRDS does not provide details on practices of dialysis treatment after incident dialysis, and thus, analyses of detailed clinical monitoring may have to rely on dialysis provider registries or other similar efforts.

Our study provides a valuable contribution to managing patients with AKI, potentially guiding the implementation of personalized treatment strategies. The scoring system offers a means of AKI prognostication that could assist clinicians in decision-making on management, monitoring protocols and follow-up procedures. The results from our study provide an impetus for several directions in future research. It is important to validate the AKI recovery scores in external datasets and in other AKI causes beyond ATN to increase generalizability and clinical applicability. Advocacy for improvements in USRDS data collection in the CMS-2728 form, particularly on AKI data, is also crucial. Additionally, we need to prioritize the assessment of patient-level information and the development of interventions tailored to the recovery risk profile. By addressing these research directions, we can aim to support more effective care for patients with AKI.

## Data Availability

The data underlying this article are available in the article.
