# Correction to: A clinical score to predict recovery in end-stage kidney disease due to acute kidney injury

**DOI:** 10.1093/ckj/sfaf299

**Published:** 2025-10-01

This is a **correction** to:

Silvi Shah, Jia H Ng, Anthony C Leonard, Kathleen Harrison, Karthikeyan Meganathan, Annette L Christianson, Charuhas V Thakar, A clinical score to predict recovery in end-stage kidney disease due to acute kidney injury, Clinical Kidney Journal, Volume 17, Issue 5, May 2024, sfae085, https://doi.org/10.1093/ckj/sfae085

Per Centers for Medicare and Medicaid policy cell suppression policy, the authors are required to suppress the cell that displays of individuals from 1 to 10. We have revised the Figures 1 and 2 accordingly.

**Figure 1:**
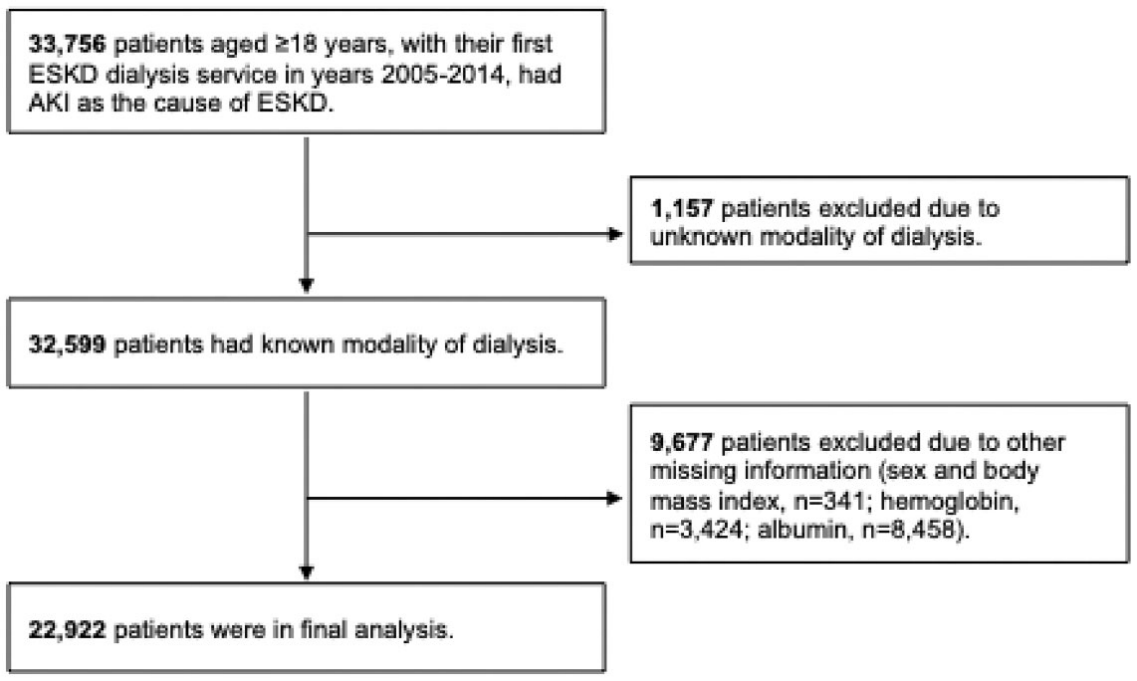
Study cohort derivation.

**Figure 2:**
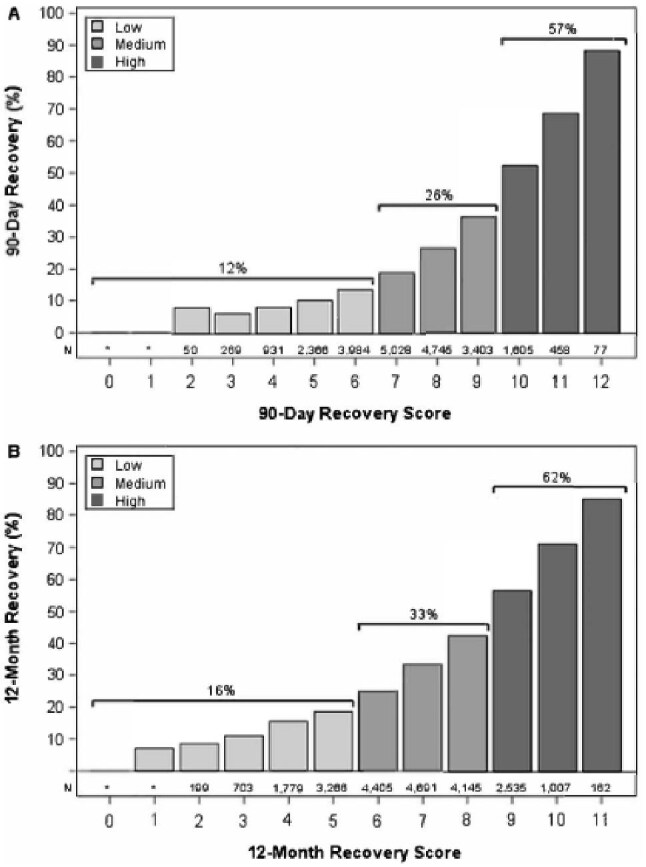
Recovery rates of patients at different recovery score levels, with total number of patients at each level, and with collapses into low, medium, and high-risk groups, for (A) recovery within 90-days post-dialysis initiation, and (B) recovery within 12-months post-dialysis initiation.

The published article has been updated.

29 January 2026 This correction notice has been amended to note that Figures 1 and 2 have been updated in the published article.

